# Supplementary material for: Enhancement of diterpenoid steviol glycosides by co-overexpressing SrKO and SrUGT76G1 genes in Stevia rebaudiana Bertoni
Source: PLoS One. 2023 Feb 6;18(2):e0260085. doi: 10.1371/journal.pone.0260085 (PMC9901802; doi:10.1371/journal.pone.0260085)
Supplement: S1 Raw images — (PDF) [file pone.0260085.s001.pdf]

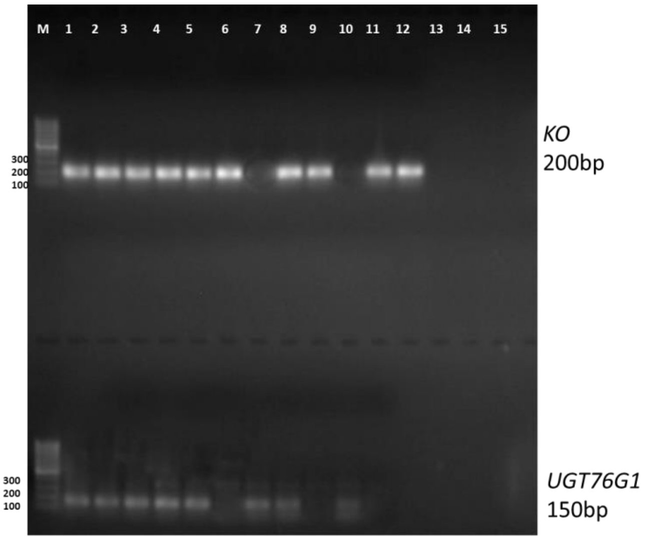

Fig. 3. Transformation confirmation by visualizing the desired amplicons on agrose gel electrophoresis.

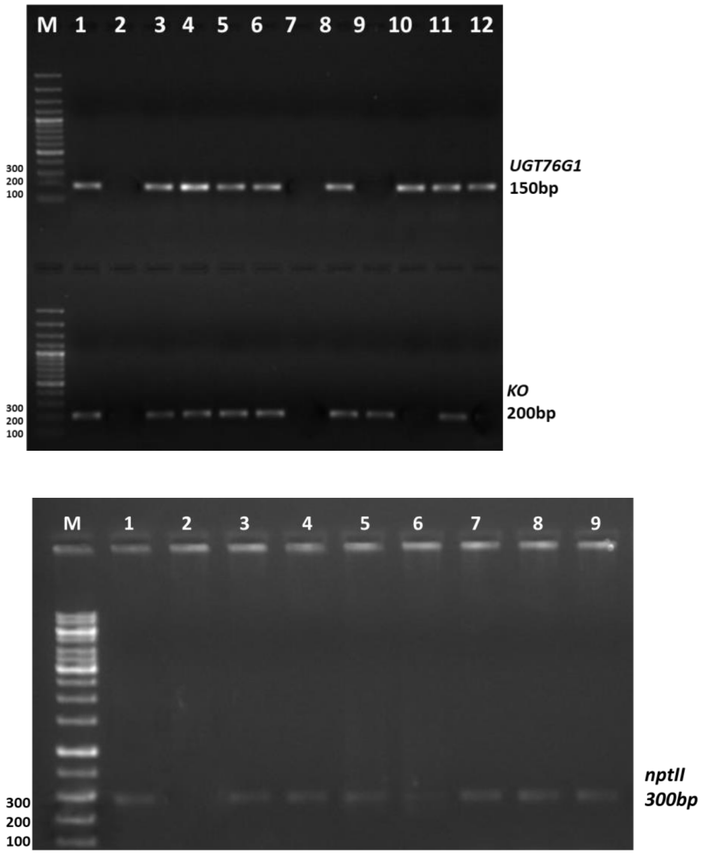

Fig. 6: A: Confirmation of putative transgenic lines by amplifying the *UGT76G1* and *KO* specific primers. Out of 10 transgenic lines, only 7 were positive for both the genes, B: transgenic lines which were positive for both the genes were further confirmed by the presence of *nptII* gene. M= 100b ladder (Fermantas) Lan 1= positive control, Lane 2= non-transformed control, Lines for *nptII* were selected for further analysis.
