# Supplementary material for: Enhancement of diterpenoid steviol glycosides by co-overexpressing SrKO and SrUGT76G1 genes in Stevia rebaudiana Bertoni
Source: PLoS One. 2023 Feb 6;18(2):e0260085. doi: 10.1371/journal.pone.0260085 (PMC9901802; doi:10.1371/journal.pone.0260085)
Supplement: Attachment — Submitted filename: PONE-D 21 11562_Reviewer comments.docx [file pone.0260085.s002.docx]

**Enhancement of diterpenoid steviol glycosides by co-overexpressing *SrKO* and *SrUGT76G1* genes in Stevia rebaudiana Bertoni.**

**Manuscript Number: PONE-D-21-11562**

**Reviewers Comments**

**Introduction:**

Introduction part was well written but still contains mistakes related to English grammar. Improve them. Authors should give page number and line number to ease the process of revision, so that errors/mistakes can be highlighted corresponding to specific lines. Some general comments in the introduction part are as follows:

- Authors must define the acronyms MVA and MEP pathways, at their first mention in the text *such as Mevalonate and Methylerythritol Phosphate Pathways. (3^rd^ paragraph of introduction)*
- Likewise, please mention the acronym *KO*. The step in GA biosynthesis is catalyzed by ent-kaurene oxidase (KO) leading to the synthesis of…………….. (*3^rd^ paragraph of introduction).*

**Materials and Methods:**

Materials and methods section was well elaborated with scientific reasonings; however, it needs some minor improvements as indicated below:

- In subsection 2.1. Chemicals for molecular research were obtained or purchased from….. (Use scientific style of writing).
- In sub section 2.2. Rephrase this sentence “The plants were grown in plastic pots (with a diameter of 31 and a height of 27 cm) in 1:1 ratio of soil and sand in poly house at Jamia Hamdard, New Delhi, India under controlled conditions, in an artificial climate chamber programmed for at 25°C/16°C 16 h/8 h day/night environment with a light intensity of 300 μmol/m2s-1 and relative humidity, 60%”
- Space should be given between a numerical value and unit like 100 mg. Please follow the Journal style for further rectification.
- *GFP* should be in capital letters*.* Please replace this in the complete manuscript.
- Why 2.4 and 2.5 subsections shared same heading “transient transformation”??
- It is mentioned in the text 1:1 ratio, which is wrong. The symbol “ : ” represents ratio, therefore no need to say 1:1 ratio. Remove the word ratio in the text.

**Results:**

This section needs improvements in following aspects as listed below:

- CAMVS35/*SrKO ??* Is it CAMVS35 or CAMV 35S? In figure the authors have mentioned CAMV 35S whereas in text it is written CAMVS35. Please use the similar and standard format in the whole manuscript to avoid confusion (see section 3.1 and 3.3 etc..)
- Authors claimed that “*The transformed leaves were confirmed by visualising the leaves under Leica TCS SP2 confocal microscope equipped with long-lasting solid-state lasers (Leica Microsystems, Germany*), BUT NO GFP visualization of transiently expressed plant tissues has been given in the form of images in the manuscript? Please elaborate?? If GFP has been visualized through microscope, it must follow by proper images. Authors must give present appropriate images of GFP visualization (sec 3.1)
- Principal component analysis (PCA) of the stevioside and rebaudioside-A gave a clear cluster pattern (Tag this line with a relevant figure?) (sec 3.1)
- Relevant figures and tables should be mentioned and tagged in section 3.1. The results of increase and decrease of quantification studies and co expression results have been discussed without mentioning pertinent figures.
- Section 3.4 gave most of the details about How the morphological analysis of shoots and leaves were carried out. This is specifically a part of materials and methods section. Please focus on the results obtained in this section.
- The result presented in Fig. 5 does not correlate with these lines in the text (In the transformed leaf, ***Sr****UBQ10* has a minimum average Cq value of 15.62, while *SrSAND* has a maximum average Cq value of 22.83). Please check it again and took help of statistician.
- Fig.13: Correlation table shows the high correlation between steviol and rebaudioside A is not present in the manuscript.

**Discussion:**

In this section, please give more reasonings about why the co-overexpression of both genes resulted in decrease of stevioside content, whereas increases RebA amounts in comparison to individual expression studies. Since, no change in RebA to stevioside ratio was observed in overexpression of SrKO. Support your results by giving reasonings and explanations from the biosynthetic pathways of both genes involved or their homologs in other plants.

**General Reviewers Comments:**

Improve English grammar and sentence structure of the manuscript. Make all necessary corrections as indicated above in each section of the manuscript. Keeping in view the significance of stevia plant and originality of research conducted by the researchers, the study holds significance and is worthy enough to be accepted in the Journal after a major revision.
