# Supplementary material for: Enhancement of diterpenoid steviol glycosides by co-overexpressing SrKO and SrUGT76G1 genes in Stevia rebaudiana Bertoni
Source: PLoS One. 2023 Feb 6;18(2):e0260085. doi: 10.1371/journal.pone.0260085 (PMC9901802; doi:10.1371/journal.pone.0260085)
Supplement: Attachment — Submitted filename: response to the reviewers.docx [file pone.0260085.s003.docx]

**Dear Editor,**

**Ploseone**

Thank you for giving us the opportunity to submit a revised draft of our manuscript titled

**“Enhancement of diterpenoid steviol glycosides by co-overexpressing *SrKO* and *SrUGT76G1* genes in Stevia rebaudiana Bertoni. Manuscript Number: PONE-D-21-11562”.** We truly appreciate the time and effort that you and reviewers have dedicated to providing your valuable feedback on our manuscript. We are grateful to the reviewers for their insightful comments on our paper. We have incorporated changes suggested by the reviewers. We have highlighted the changes in the manuscript.

Here are the point to point response to the reviewers’ comments and concerns.

**Reviewers Comments**

**Introduction:**

*Introduction part was well written but still contains mistakes related to English grammar. Improve them. Authors should give page number and line number to ease the process of revision, so that errors/mistakes can be highlighted corresponding to specific lines. Some general comments in the introduction part are as follows:*

**Response: As suggested english grammar has been checked by experts and by online available software Premium ProwritingAid.**

- *Authors must define the acronyms MVA and MEP pathways, at their first mention in the text such as Mevalonate and Methylerythritol Phosphate Pathways. (3^rd^ paragraph of introduction)*

**Response: *Acronyms have been properly defined in the manuscript in page 1, line 17.***

- Likewise, please mention the acronym *KO*. The step in GA biosynthesis is catalyzed by ent-kaurene oxidase (KO) leading to the synthesis of…………….. (*3^rd^ paragraph of introduction).*

**Response: Acronyms have been properly defined in the manuscript in page 1, line 19**.

**Materials and Methods:**

Materials and methods section was well elaborated with scientific reasonings; however, it needs some minor improvements as indicated below:

- *In subsection 2.1. Chemicals for molecular research were obtained or purchased from….. (Use scientific style of writing).*

**Response: We have changed the sentence in a more scientific style as suggested on page 2, line 56-57.**

- *In sub section 2.2. Rephrase this sentence “The plants were grown in plastic pots (with a diameter of 31 and a height of 27 cm) in 1:1 ratio of soil and sand in poly house at Jamia Hamdard, New Delhi, India under controlled conditions, in an artificial climate chamber programmed for at 25°C/16°C 16 h/8 h day/night environment with a light intensity of 300 μmol/m2s-1 and relative humidity, 60%”*

**Response: We have rephrased the sentence in a more scientific style as suggested on page 3, line 73-75.**

- *Space should be given between a numerical value and unit like 100 mg. Please follow the Journal style for further rectification.*

**Response: Space has been incorporated as suggested in page 3, line 77.**

- *GFP should be in capital letters. Please replace this in the complete manuscript.*

**Response: We have replaced gfp by *GFP* at all places in the manuscript.**

- *Why 2.4 and 2.5 subsections shared same heading “transient transformation”??*

**Response: Thank you for pointing this out, as suggested we have changed the name of the subsection 2.4.**

- *It is mentioned in the text 1:1 ratio, which is wrong. The symbol “ : ” represents ratio, therefore no need to say 1:1 ratio. Remove the word ratio in the text.*

**Response:** **we have corrected it as suggested.**

**Results:**

This section needs improvements in following aspects as listed below:

- *CAMVS35/SrKO ?? Is it CAMVS35 or CAMV 35S? In figure the authors have mentioned CAMV 35S whereas in text it is written CAMVS35. Please use the similar and standard format in the whole manuscript to avoid confusion (see section 3.1 and 3.3 etc..)*

**Response:** **We have corrected it as suggested.**

- *Authors claimed that “The transformed leaves were confirmed by visualising the leaves under Leica TCS SP2 confocal microscope equipped with long-lasting solid-state lasers (Leica Microsystems, Germany), BUT NO GFP visualization of transiently expressed plant tissues has been given in the form of images in the manuscript? Please elaborate?? If GFP has been visualized through microscope, it must follow by proper images. Authors must give present appropriate images of GFP visualization (sec 3.1)*

**Response: We have incorporate the** Figures of GFP visualization in the manuscript.

- *Principal component analysis (PCA) of the stevioside and rebaudioside-A gave a clear cluster pattern (Tag this line with a relevant figure?) (sec 3.1)*

**Response: Thank you for pointing this sentence out, the sentence was mistakenly incorporated here and has been removed.**

- *Relevant figures and tables should be mentioned and tagged in section 3.1. The results of increase and decrease of quantification studies and co expression results have been discussed without mentioning pertinent figures.*

**Response: Figures have been incorporated as suggested on page 8, line 223.**

- Section 3.4 gave most of the details about How the morphological analysis of shoots and leaves were carried out. This is specifically a part of materials and methods section. Please focus on the results obtained in this section.

**Response:** **Modified as suggested at page 9, line 272.**

- *The result presented in Fig. 5 does not correlate with these lines in the text (In the transformed leaf,* ***Sr****UBQ10 has a minimum average Cq value of 15.62, while SrSAND has a maximum average Cq value of 22.83). Please check it again and took help of statistician.*

**Response: Cq value is the average of all the samples.**

- *Fig.13: Correlation table shows the high correlation between steviol and rebaudioside A is not present in the manuscript.*

**Response: Correlation table has been incorporated in the manuscript.**

**Discussion:**

*In this section, please give more reasonings about why the co-overexpression of both genes resulted in decrease of stevioside content, whereas increases RebA amounts in comparison to individual expression studies. Since, no change in RebA to stevioside ratio was observed in overexpression of SrKO. Support your results by giving reasonings and explanations from the biosynthetic pathways of both genes involved or their homologs in other plants.*

**Response:** **Reasoning has been given in the manuscript as suggested on page 19, line 502 and 510.**

**General Reviewers Comments:**

*Improve English grammar and sentence structure of the manuscript. Make all necessary corrections as indicated above in each section of the manuscript. Keeping in view the significance of stevia plant and originality of research conducted by the researchers, the study holds significance and is worthy enough to be accepted in the Journal after a major revision.*

**Response: We have made all the changes as suggested, we look forward to hearing from you in due time regarding our submission and to respond to any further questions and comments you may have.**

**Sincerely,**

**Dr. Nazima Nasrullah**
